# Ex Vivo Shear-Wave Elastography for Intraoperative Prediction of Axillary Lymph Node Metastasis in Breast Cancer

**DOI:** 10.3390/medicina62091730

**Published:** 2026-09-08

**Authors:** Süleyman Özkan Aksoy, Işıl Başara Akın, Gökçe Kıran Kazancı, Merih Güray Durak, Canan Altay, Zekai Serhan Derici, Cihan Ağalar, Berke Manoğlu, Muhammet Berkay Sakaoğlu, Erhan Tükel, Pınar Balcı, Ali İbrahim Sevinç

**Affiliations:** 1 Department of General Surgery, Faculty of Medicine, Dokuz Eylül University, İzmir 35340, Turkey; gokcekiran28@gmail.com (G.K.K.); serhan.derici@deu.edu.tr (Z.S.D.);; 2 Department of Radiology, Faculty of Medicine, Dokuz Eylül University, İzmir 35340, Turkey; slbasara@yahoo.com (I.B.A.);; 3 Department of Pathology, Faculty of Medicine, Dokuz Eylül University, İzmir 35340, Turkey

**Keywords:** breast cancer, shear-wave elastography, sentinel lymph node, ex vivo, intraoperative staging, lymph node metastasis

## Abstract

*Background and Objectives:* Intraoperative sentinel lymph node (SLN) assessment is a pivotal step in early-stage breast cancer surgery. Frozen section (FS) analysis remains a widely utilised technique; however, its limitations, particularly in the detection of micrometastases, are well-documented. The process is both time-consuming and costly. The objective of this study was to investigate the potential of ex vivo shear-wave elastography (SWE) as a rapid and reproducible method for quantifying lymph node stiffness and predicting metastatic involvement. *Materials and Methods:* Between January 2022 and January 2024, 100 patients with biopsy-confirmed invasive breast cancer undergoing SLNB were enrolled in the study. A total of 384 excised axillary lymph nodes were subjected to ex vivo SWE immediately following excision, prior to the standard histopathological procedure. The mean stiffness (Emean) values were recorded, and the diagnostic performance was evaluated with the use of ROC analysis. *Results:* Of the 384 nodes examined, 45 (11.6%) were found to have metastases on final histopathology. It was demonstrated that Emean exhibited excellent discriminatory power, with an Area Under the Curve (AUC) value of 0.957. At the optimal cut-off of 28 kPa, the sensitivity and specificity levels were recorded as 91% and 87%, respectively. *Conclusions:* The findings of this study indicate that ex vivo SWE is a highly accurate and reproducible method for identifying metastatic sentinel lymph nodes. The technique has the potential to complement or selectively replace intraoperative FS, thereby reducing unnecessary tissue processing. The logical next step is multicentre validation.

## 1. Introduction

Breast cancer is the most prevalent form of cancer among the global female population. Axillary lymph node (LN) status has been identified as the strongest predictor of recurrence and long-term survival [1]. It is therefore imperative to ensure that the staging is accurate from the outset, as this directly influences crucial decisions regarding the extent of axillary surgery and adjuvant treatment. For patients who are clinically node-negative (cN0), the sentinel lymph node biopsy (SLNB) is now the standard of care that is endorsed by the guidelines [1,2].

However, the intraoperative methods employed for interrogation of these sentinel nodes are all associated with well-recognised shortcomings. Frozen section (FS), touch imprint cytology (TIC), and one-step nucleic acid amplification (OSNA) each demand dedicated resources, add operative time, and—crucially—miss a meaningful proportion of micrometastases [3,4,5]. Across published series, sensitivity for sub-2 mm deposits can fall dramatically, leaving surgeons to rely on permanent sections and, in some cases, second operations [3,5]. There is a genuine clinical need for a solution that is faster, more cost-effective, and less dependent on intraoperative pathology infrastructure.

Shear-wave elastography (SWE) is a method of measuring tissue stiffness that is non-invasive and does not involve the use of radiation. This is achieved by quantifying the propagation speed of mechanically induced shear waves. The underlying principle is uncomplicated: malignant lymph nodes exhibit increased stiffness relative to reactive or benign nodes, and SWE quantifies this difference numerically [6,7,8,9]. A 2022 systematic review confirmed that Emax and Emean show promising sensitivity and specificity for LN malignancy, with diagnostically strong performance across multiple body sites [10].

The ex vivo environment is a particularly attractive setting for elastography. In vivo measurements are compromised by probe pressure artefacts, patient movement, and the depth of axillary tissue; ex vivo, those confounders are eliminated [11,12,13,14]. The node is positioned within a gel bath, with access from all sides, under conditions that are both controlled and reproducible [13,15].

Two studies by Bae and colleagues laid important groundwork. In a 228-node ex vivo series, metastatic nodes demonstrated significantly higher levels of stiffness and size, with AUC values ranging from 0.79 to 0.83 [14]. The subsequent nomogram developed by the researchers combined node size with SWE elasticity. This combination achieved an area under the curve (AUC) of 0.856 and maintained this at 0.79 in a validation cohort. This suggests that there is a possibility of replacing intraoperative FS with an ultrasound-only workflow in selected patients [16]. However, it is imperative to exercise caution when interpreting these findings, as a 168-node ex vivo SLN study has revealed no substantial SWE velocity disparities between metastatic and benign nodes [17]. It is important to note that the evidence, in summary, appears to be promising, albeit contested.

The present diagnostic study was thus designed to evaluate 384 axillary lymph nodes from 100 consecutive breast cancer patients using ex vivo SWE, with a view to ascertaining whether quantitative elasticity parameters can reliably distinguish metastatic from benign nodes. Furthermore, the study will explore the practical role of this approach in intraoperative axillary staging.

## 2. Materials and Methods

### 2.1. Study Design and Participants

This study was conducted at the Department of General Surgery, Dokuz Eylül University Faculty of Medicine, between January 2022 and January 2024. Consecutive patients with biopsy-confirmed breast cancer scheduled for sentinel lymph node biopsy (SLNB) as part of standard multidisciplinary care were considered for enrolment.

### 2.2. Eligibility

The study included women aged 18 years and over who were deemed to have clinically node-negative axillary status (cN0) on physical examination and preoperative ultrasound. The absence of sonomorphological red flags, such as cortical thickening, hilum loss, or rounded contours, indicated a node-negative status. Patients with a history of ipsilateral axillary surgery or radiotherapy were excluded from the study.

Furthermore, the study excluded men, pregnant or lactating women, cases yielding poor-quality elastography images (signal dropout, air-bubble artefacts, or inadequate gel contact), and patients who had received neoadjuvant chemotherapy (NAC). The NAC exclusion was deliberate, as it is well established that cytotoxic treatment alters the mechanical properties of lymphoid tissue in ways that could confound stiffness measurements.

### 2.3. Ethics and Consent

The protocol was approved by the local institutional ethics committee and conducted throughout in accordance with the Declaration of Helsinki. The management of the data was undertaken in strict accordance with the protocols of confidentiality.

### 2.4. Sentinel Lymph Node Biopsy

The present study employed 99mTc-nanocolloid (Eczacıbaşı Monrol Nuclear Products Co., Istanbul, Türkiye) and/or blue dye [methylene blue (Blumet; VEM İlaç, Ankara, Türkiye) or patent blue (Faculty of Pharmacy, Istanbul University, Istanbul, Türkiye)] for SLN mapping. Nodes exhibiting the most intense gamma-probe activity or blue dye uptake were then excised. All fresh specimens were transferred without fixation to the radiology unit for ex vivo evaluation before entering the pathology workflow.

### 2.5. Ex Vivo Ultrasound and SWE Protocol

All B-mode ultrasonographic and elastographic evaluations were performed using a Philips EPIQ Elite ultrasound system (Philips Healthcare, Bothell, WA, USA) equipped with an eL18-4 linear probe. To ensure optimal acoustic coupling, a sufficient layer of bubble-free ultrasound gel was applied over each target area. Each examination commenced with a standard B-mode assessment, encompassing the evaluation of axial dimensions, cortical thickness, and hilum architecture, followed by the 2D-SWE investigation using the ElastQ Imaging module.

**Figure 1:**
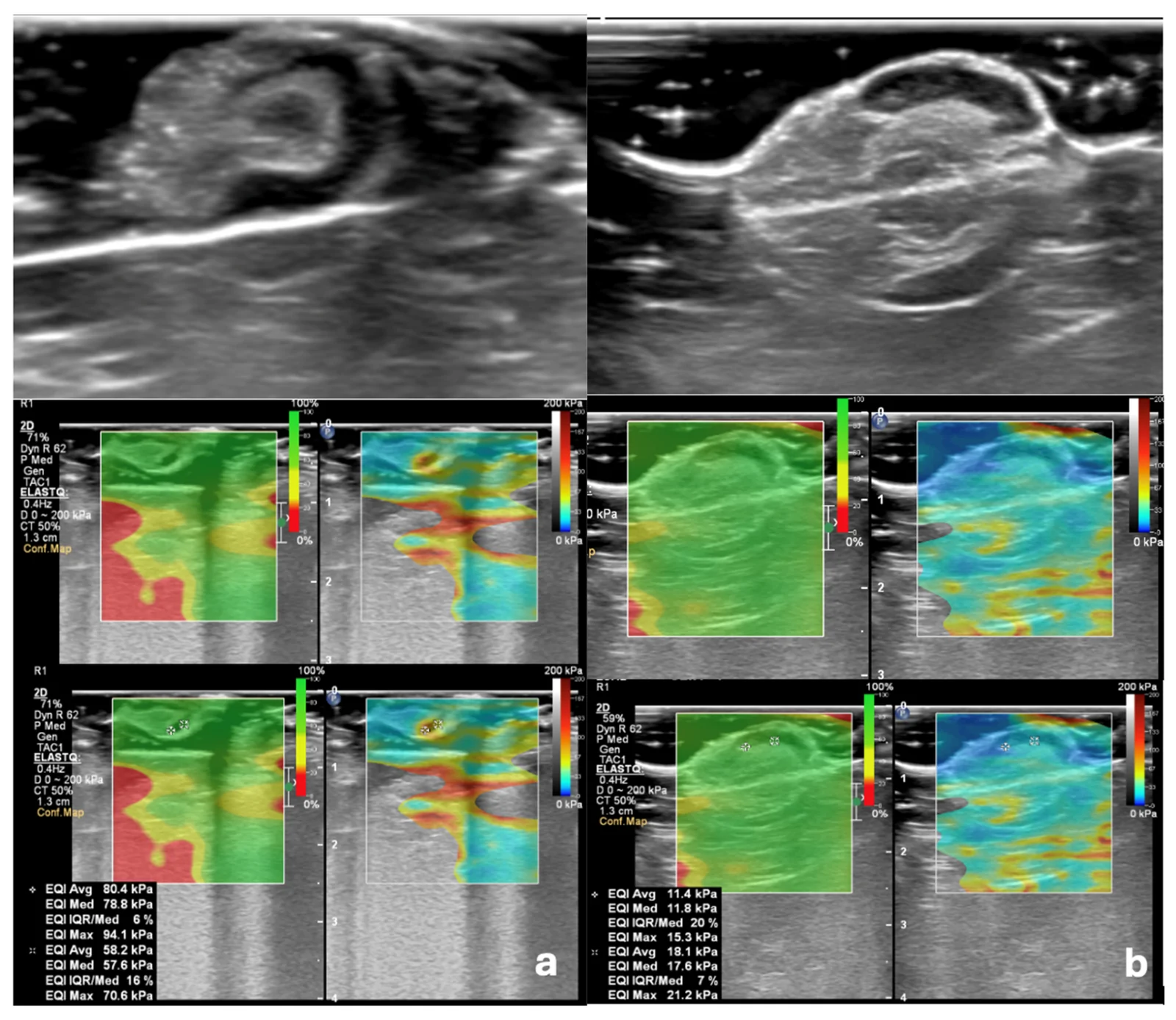
(**a**). Sentinel lymph node, malignant diagnosis. Mean elasticity values: 80.4–58.2 kPa. (**b**). Sentinel lymph node, benign diagnosis. Mean elasticity values: 11.4–17.6 kPa.

During SWE acquisition, the system’s integrated intelligent analysis tool (Confidence Map) was activated, and data collection was restricted to homogeneous tissue areas with high propagation confidence (color-coded as green) (Figure 1a,b). Quantitative elasticity values were obtained using region of interest (ROI) windows placed within the stiffest visible areas of the lymph node cortex. The mean stiffness values were recorded and expressed in kilopascals (kPa).

### 2.6. Histopathological Reference Standard

Following the evaluation of SWE, the nodes were subsequently dispatched to the pathology department. Sections were cut at 2–3 mm intervals, paraffin-embedded, and stained with haematoxylin and eosin (H&E). The pathologists were unaware of the elastography data. The deposits were then classified according to their size, with metastases defined as macrometastasis (>2 mm), micrometastasis (0.2–2 mm), or isolated tumour cells (<0.2 mm, ITCs).

### 2.7. Statistical Analysis

The following metrics are employed for continuous variables: mean ± standard deviation or median (interquartile range). Categorical variables are reported as frequency (%). The comparison of elasticity parameters between benign and metastatic nodes was conducted utilising Student’s *t*-test or the Mann–Whitney U test, depending on the distribution of the data. The diagnostic performance was assessed by ROC curve analysis, and the Youden index was utilised to determine the optimal cut-off values.

Because multiple lymph nodes were obtained from individual patients, an additional patient-cluster bootstrap sensitivity analysis was performed to account for within-patient correlation. Patients, rather than individual lymph nodes, were used as the resampling unit, with all lymph nodes from each selected patient retained together within each bootstrap sample. A total of 10,000 cluster-bootstrap replicates were used to obtain 95% confidence intervals for the AUC, sensitivity, and specificity. Diagnostic performance at the Emean threshold of 28 kPa was also evaluated using this cluster-adjusted approach.

During the preparation of this manuscript, a generative artificial intelligence tool (Consensus.app (Consensus NLP, Inc., San Francisco, CA, USA; web-based application, available at https://consensus.app/, accessed on 5 May 2026)) was used solely to assist with language editing, grammatical revision, and clarity of phrasing in the English text. The AI tool was not involved in data collection, analysis, interpretation, or the formulation of scientific conclusions. All content was reviewed and edited by the authors, who take full responsibility for the accuracy and integrity of the published work.

## 3. Results

### 3.1. Patient Characteristics

The present study enrolled a total of 100 patients diagnosed with invasive breast cancer. The mean age of the subjects was 59.64 ± 12.30 years (range 31–87 years). The majority of patients were postmenopausal (80%), while 20% were pre- or perimenopausal. The complete clinicopathological details are presented in Table 1.

Tumour staging: 62 patients were classified as T1, 36 as T2, and 2 as T3. Nodal staging at the time of surgery: The distribution of cases is as follows: no. 0 in 68, no. 1 in 29, no. 2 in 3. With regard to histological grade, grade 1 was observed in 21 cases, grade 2 in 64, and grade 3 in 15. Immunohistochemical analysis revealed that 91 patients (91%) were ER-positive, 80 (80%) were PR-positive, and 13 (13%) were HER2-positive. By molecular subtype: The distribution of the samples is as follows: 54 Luminal A, 31 Luminal B, 13 HER2-positive, and 4 triple-negative.

### 3.2. Findings Relating to Lymph Nodes

The clinical–pathological evaluation of patients with metastatic lymph nodes (N+) and those without metastasis (N−) revealed statistically significant differences in pathological tumor size (*p* = 0.012), histological grade (*p* = 0.040), and HER2 receptor status (*p* = 0.028).

### 3.3. Diagnostic Accuracy

**Figure 2:**
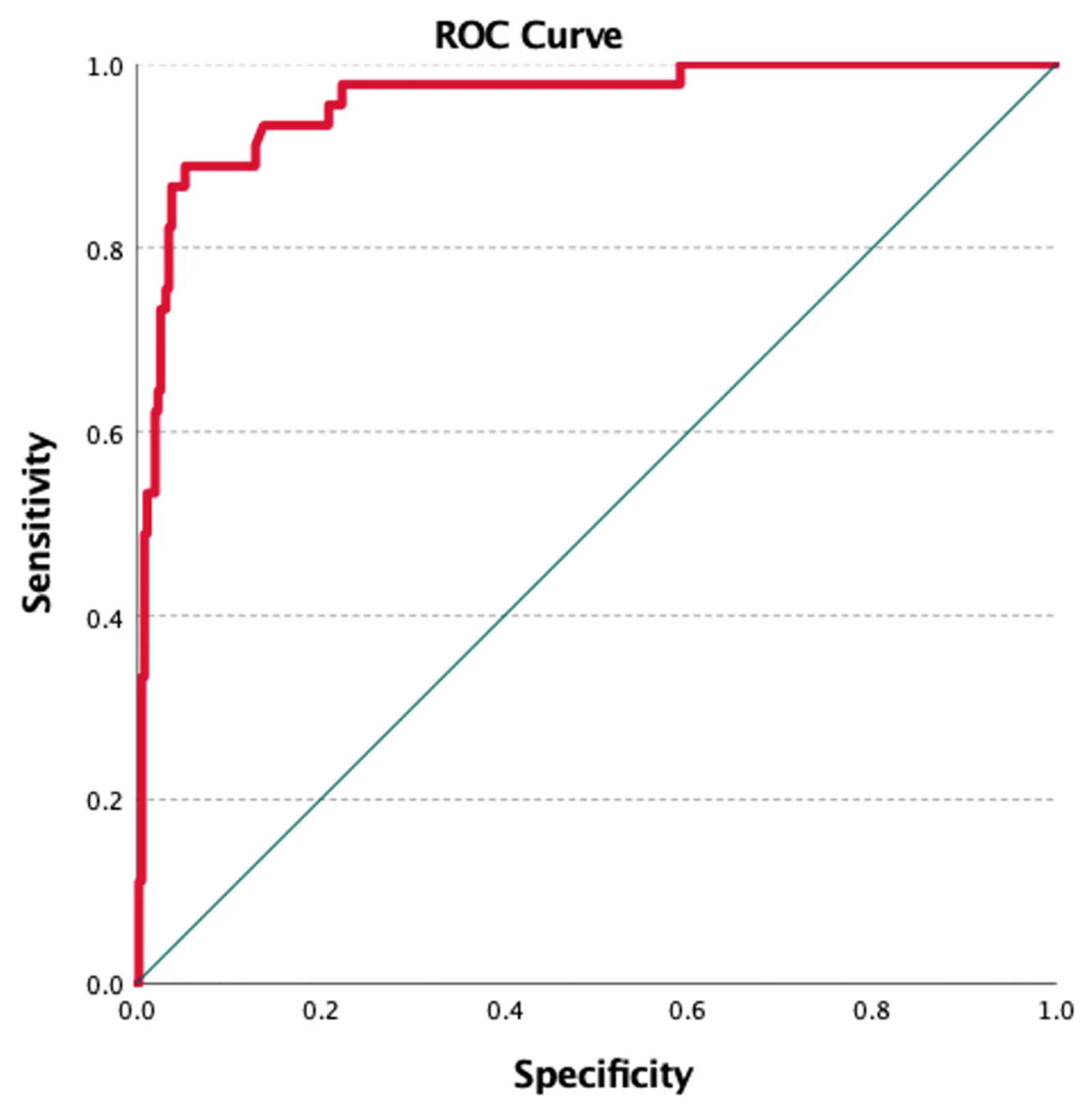
ROC curve of Emean for predicting sentinel lymph node metastasis using ex vivo shear-wave elastography. The AUC was 0.957, with an optimal cut-off value of 28 kPa, yielding 91% sensitivity and 87% specificity.

The ROC curve analysis for individual elasticity parameters is demonstrated in Figure 2. It is evident that Emean demonstrated the most robust performance, exhibiting an Area Under the Curve (AUC) value of 0.957 (95% CI: 0.927–0.987, *p* < 0.001). This outcome is regarded as exceptional in accordance with established criteria. At the Youden-optimal cut-off of 28 kPa, the sensitivity was 91%, and the specificity was 87% (Table 2). The ROC curve provides a visual representation of the relationship between stiffness and metastatic nodes, demonstrating the efficacy of a single quantitative parameter as a robust predictor of metastasis in this cohort.

To account for the potential correlation among multiple lymph nodes obtained from the same patient, a patient-cluster bootstrap sensitivity analysis was additionally performed. The cluster-adjusted AUC for Emean was 0.953 (95% CI: 0.913–0.981)**.** At the 28-kPa threshold, sensitivity was 88.9% (95% CI: 77.5–97.4%) and specificity was 86.7% (95% CI: 81.9–91.2%). These estimates were consistent with the primary node-level analysis, indicating that adjustment for within-patient clustering did not materially alter the diagnostic performance of Emean.

## 4. Discussion

The principal finding of this study is unambiguous. Ex vivo SWE was shown to predict sentinel lymph node metastasis in breast cancer with an area under the curve (AUC) of 0.957, a sensitivity of 91%, and a specificity of 87% at a 28 kPa threshold. The numbers thus obtained place the technique in a range that is not only statistically impressive but clinically meaningful. Specifically, the performance of a tool in one session does not necessarily translate to its efficacy in another, thus limiting its surgical application.

A comparison with frozen section is warranted. FS is a rapid and precise method for the detection of macrometastases; however, its sensitivity to micrometastatic disease remains a persistent weak point. A substantial body of research, encompassing multiple series and meta-analyses, has documented a notable increase in false-negative rates when deposit size falls below 2 mm [18,19]. When these requirements are given due consideration, including the necessity for a cryostat, an on-call pathologist, and a defined slice-and-freeze time window, it becomes evident that FS is a resource-intensive procedure, particularly for centres with limited intraoperative pathology capacity. It is not being contended that FS should be eschewed; its specificity with regard to macrometastases is challenging to emulate. Nevertheless, conducting a cost–benefit analysis, particularly for lower-risk patients with small primary tumours, is a worthwhile exercise.

A preliminary investigation of the extant ex vivo SWE literature was conducted prior to the commencement of the presented study. Bae et al. reported an AUC range of 0.79 to 0.83 across 228 nodes [14] and subsequently constructed a nomogram achieving an accuracy of 0.856, with a maintained value of 0.79 in the validation process [16]. These are real-world numbers with which a clinician would be familiar. Kilic et al. also identified significant disparities in stiffness between metastatic and benign SLNs [17]. In contrast, Togawa et al. conducted a study on 168 SLNs, which revealed no substantial SWE velocity disparities, thereby under-scoring the notion that promising pilot data does not invariably translate into replicable outcomes [20]. The present study utilised a cohort of 384 nodes, and histopathologically verified, constituting the most extensive ex vivo SWE dataset in this setting to date. The observed AUC (0.957) is higher than in previous series, which may be indicative of the standardised ex vivo protocol, the gel-immersion technique, and the deliberate exclusion of NAC-treated patients.

These findings suggest a potential role for ex vivo SWE as a complementary triage tool rather than as a replacement for frozen-section analysis. However, the 28-kPa threshold identified in the present study was derived and evaluated within the same single-centre cohort and should therefore be regarded as exploratory. Although the observed diagnostic performance supports further investigation of Emean-based risk stratification, the present data do not justify withholding intraoperative histopathological assessment on the basis of this threshold alone. Prospective external validation in independent, preferably multicentre cohorts is required before a specific SWE threshold can be incorporated into clinical decision-making.

The present study acknowledges the limitations of the research. Firstly, it should be noted that this is a single-centre study; as a result, the generalisability of the results is uncertain until external validation is available. Secondly, diagnostic performance could not be evaluated separately for macrometastases, micrometastases, and isolated tumor cells. This is particularly relevant because small metastatic deposits represent an important limitation of intraoperative frozen-section analysis. Therefore, the present findings should not be interpreted as demonstrating superior performance of ex vivo SWE specifically for micrometastatic disease or isolated tumor cells. Thirdly, we did not formally record the time added by ex vivo SWE to the operative workflow, which is a practical question that any adopting centre will ask. These gaps delineate the agenda for the subsequent iteration of this work.

In order to advance this research, it would be advantageous to conduct a multicentre trial that randomises or matches patients to SWE-guided versus standard FS-based intraoperative staging. It is imperative that this trial encompasses not only evaluation of diagnostic accuracy, but also a comprehensive analysis of secondary surgical interventions, operative time, financial costs, and long-term oncological outcomes [16]. Should SWE-based triage prove to be both safe and cost-effective in this particular setting, it has the potential to effect a paradigm shift in the manner in which intraoperative axillary staging is conducted. This would not result in the obsolescence of FS, but rather in its reservation for cases in which its application is truly necessary.

## 5. Conclusions

Ex vivo SWE has been shown to be a valuable tool in identifying metastatic sentinel lymph nodes in breast cancer patients. This method had an AUC of 0.957, with a sensitivity of 91% and a specificity of 87% at the 28 kPa threshold. These results suggest that ex vivo SWE could be used as a complementary ultrasound-based triage tool to reduce the need for intraoperative frozen section analysis. However, larger multicentre studies are needed to validate these results.

## Figures and Tables

**Table 1:** Clinicopathological characteristics of the patients.

Characteristic	Total	Benign	Malign	*p*
Age (years), mean ± SD	59.64 ± 12.30	60.8 ± 12.5	57.1± 11.5	0.167
Menopausal status, *n* (%)				
Pre/perimenopausal	20 (20)	11 (16.2)	9 (28.1)	0.163
Postmenopausal	80 (80)	57(83.8)	23 (71.9)	
Tumour stage (T), *n* (%)				
T1	62 (62)	46(67.6)	16 (50)	0.412
T2	36 (36)	22(32.4)	14 (43.7	
T3	2 (2)	-	2 (6.3)	
Histological grade, *n* (%)				
Grade 1	21 (21)	16 (23.8)	5 (15.6)	0.040
Grade 2	64 (64)	41 (60.2)	23 (71.8)	
Grade 3	15 (15)	11 16.1)	4 (12.5)	
ER status, *n* (%)				
Positive	91 (91)	60 (88.2)	31 (96.8)	0.430
Negative	9 (9)	8 (11.8)	1 (3.2)	
PR status, *n* (%)				
Positive	80 (80)	52 (76.4)	27(84.3)	0.453
Negative	20 (20)	15 (24.6)	5 (15.7)	
HER2 status, *n* (%)				
Positive	13 (13)	10	3	0.028
Negative	87 (87)	58	29	

ER, oestrogen receptor; PR, progesterone receptor; HER2, human epidermal growth factor receptor 2; SD, standard deviation.

**Table 2:** Diagnostic performance of ex vivo SWE parameters for predicting sentinel lymph node metastasis.

Parameter	AUC (95% CI)	Optimal Cut-Off (kPa)	Sensitivity (%)	Specificity (%)	*p*
Emean	0.957 (0.927–0.987)	28	91	87	<0.001

AUC, area under the receiver operating characteristic curve; CI, confidence interval; SWE, shear wave elastography.

## Data Availability

The datasets supporting the conclusions of this article are included within the article (and its additional files).

## Abbreviations

The following abbreviations are used in this manuscript:

SLN	Sentinel lymph node
FS	Frozen section
SWE	Shear-wave elastography
AUC	Area under the curve
LN	Lymph node
SLNB	Sentinel lymph node biopsy
cN0	Clinically node-negative
TIC	Touch imprint cytology
OSNA	One-step nucleic acid amplification
NAC	Neoadjuvant chemotherapy
ROI	Region of interest
H&E	Haematoxylin and eosin
ITC	Isolated tumour cell
SD	Standard deviation
ROC	Receiver operating characteristic
IQR	Interquartile range
ER	Oestrogen receptor
PR	Progesterone receptor
HER2	Human epidermal growth factor receptor 2
kPa	Kilopascal
